# Ranking’s Abstract

**Published:** 1855-09

**Authors:** 


*Ranking's Abstract.*—We are in receipt of the 21st No. of this most valuable periodical, containing 299 pages of choice contributions to medical science. We would be gratified in being able to present a synopsis of the work and a brief review of many of the articles found upon its pages, but time and limit forbid. In *practical medicine* it contains much that is new and interesting, and in the department of *surgery*, as well as *midwifery*, the reader will find upon examination numerous items of deep interest and concern. It is published by Lindsay & Blackiston, Philadelphia, an establishment whence proceeds, from time to time, large and valuable additions to medical literature. Terms, two dollars per annum, and published half-yearly. The above mentioned firm has won golden opinions from the profession for their efforts to supply the profession of this country with so many valuable books on medicine.

